# Will It Prove a Wise Departure

**Published:** 1897-10

**Authors:** 


### WILL IT PROVE A WISE DEPARTURE?

THE issues that decided the election of the President of the U. S. V. M. A. at Nashville for the ensuing year mark a departure from the custom of according a second term in the Association, from its birth to the present time, with perhaps two exceptions—one of which we have no disposition to discuss, because it involves a deceased member; the second was under most extraordinary circumstances, and occurs only once, perhaps, in an organization's history. We refer to the decision that the President shall be elected but for one term.

As was well said by the outgoing President, he is virtually in office for one year before he can announce or outline his policy, and all his plans must, under the new rule, fall to his successor to accept and carry out as he may determine their merits. While, in common with every faithful member to Association interests, we bow to the will of the majority and rejoice that our interests in the national organization are to be confided to the hands of one worthy in every sense of the high honor he has received, and fully assured that his interest will be the welfare of all, his administration rank among the highest and best ever given by a chief officer, we still are not sure of this being a wise departure. We are not unmindful of how few are the chances for a member to become President during an average length of years of active association service, and of how fortunate we are in having so large a number of available men for this place; still the duties of this office, as our Association continues its present line of work, are onerous, and he who receives this recognition finds that his shoulders are freighted with responsibilities that grow greater and more exacting every year.

Was this position solely one of honor, as in at least one organization similar to ours, where, after many years, section work has been established—each section with its corps of officers to direct and control the work of its department—we could join with others in the belief that this was a movement in the right direction.

Again, does it not force upon us, in this departure, the decision that in the future we must have our organization divided into sections? And this, in turn, forces the query, Are we ready yet to divide into sections? Let us look at the situation for a few minutes. Scarcely ten years of active work have passed over our heads; we have barely reached five hundred in number out of possibly three thousand in the United States; we have all had a narrow enough education, to say the least; we have never had a full hundred of our members present at any one meeting; and of the last six meetings less than fifty can be counted who had been in regular attendance at each annual convention—not enough to officer properly five sections, as already indicated, and no audience for any of these to listen to the papers and discuss the questions. Again, how many of us know much about everyday practice, and nothing at all of State medicine or those wider subjects of greatest public importance? And is it not imperative for us to go to our national gatherings to get in touch with these movements, that something may be done for the common good in the community where one lives besides the advancement of one's selfish interests? With such a small attendance at our national gatherings, are we ready to divide into sections, and thus lose the value and the force of our deliberations and conclusions, as we must do when coming only from small sections of our Association?

The questions of only one term for a President and the division into sections are serious and far-reaching problems for us to well consider. And every possible outcome of these movements should be carefully weighed, lest the good work of the past ten years be endangered and our power as a scientific organization and leader of public sentiment in the veterinary sanitary world become impotent for good and barren of results. The great field of work and usefulness now so close within our grasp, gained after so many years of hardship and struggle, must not be endangered for a moment by any experimental steps or projects of uncertainty in our present numerical strength.

